# Anomalous shear band characteristics and extra-deep shock-affected zone in Zr-based bulk metallic glass treated with nanosecond laser peening

**DOI:** 10.1038/srep43948

**Published:** 2017-03-07

**Authors:** Yanpeng Wei, Guangyue Xu, Kun Zhang, Zhe Yang, Yacong Guo, Chenguang Huang, Bingchen Wei

**Affiliations:** 1 Key Laboratory for Mechanics in Fluid Solid Coupling Systems, Institute of Mechanics, Chinese Academy of Sciences, Beijing 100190, China; 2 Key Laboratory of Microgravity (National Microgravity Laboratory), Institute of Mechanics, Chinese Academy of Sciences, Beijing 100190, China; 3 China Electronics Technology Group Corporation No.38 Research Institute, Hefei 230031, China

## Abstract

The effects of nanosecond laser peening on Zr_41_Ti_14_Cu_12.5_Ni_10_Be_22.5_ metallic glass were investigated in this study. The peening treatment produced an extra-deep shock-affected zone compared to crystal metal. As opposed to the conventional shear bands, numerous arc shear bands appeared and aggregated in the vertical direction of the laser beam, forming basic units for accommodating plastic deformation. The arc shear bands exhibited short and discrete features near the surface of the material, then grew longer and fewer at deeper peened layer depths, which was closely related to the laser shock wave attenuation. An energy dissipation model was established based on Hugoniot Elastic Limit and shear band characteristics to represent the formation of an extra-deep shock-affected zone. The results presented here suggest that the bulk modification of metallic glass with a considerable affected depth is feasible. Further, they reveal that nanosecond laser peening is promising as an effective approach to tuning shear bands for improved MGs ductility.

Bulk metallic glasses (BMGs), which possess many attractive properties including high strength, elastic deformability, and corrosion resistance, have attractive potential as structural materials^1^,^2^,^3^,^4^,^5^. BMGs generally suffer from low ductility at ambient temperature, however, which limits their practical application. Shear bands (SBs) are preferential sites for further plastic flow due to structural and thermal softening^6^,^7^,^8^. The unlimited propagation of localized SBs leads to catastrophic failure along a single SB^9^,^10^,^11^.

Over the past decade, several previous researchers have attempted to tune SBs to improve BMG ductility via either microstructure modifications or surface modifications^12^,^13^,^14^,^15^,^16^,^17^,^18^,^19^,^20^,^21^,^22^. These methods were conducted with a common aim: the generation of homogeneous SB while preventing excessive localized shear-banding upon plastic deformation^23^. The microstructural inhomogeneity caused by an exogenous phase or crystallization can prevent persistent slippage on individual SBs^12^,^13^,^16^,^18^. Other methods such as defect-printing treatments^19^ cold-rolling^24^ and surface mechanical attrition treatments^25^,^26^,^27^ are also performed to generate pre-existing shear softened region that can serve as the onset and arrest of new SBs. Can homogeneous SBs with discrete distribution and controlled length be successfully prepared to prevent (or at least delay) brittle fracture?

The propagating front runs at speed close to the transverse sound velocity^7^. The total time available for SB growth and development is in the order of *t* = 10^−5^*s*^28^, i.e., is much shorter than the time window produced by conventional modifications. This sizable difference in duration between SB propagation and conventional modification allows most SBs to be propagated to their full lengths after arrest. There is a great deal of research interest in prefabricating SBs in BMGs, as the propagation of SBs is suspended instantaneously upon onset.

Shot peening has been utilized on a variety of materials for surface strengthening in recent studies, and is especially popular for BMGs^29^. The amplitude of the shock wave is high enough to cause irrecoverable deformation until the amplitude has fallen below the dynamic yield strength of the material. Compared to shot peening, nanosecond laser peening creates a much larger shock wave peak in amplitude^30^,^31^. The action time is so short (less than 100 *ns*) that SB propagation can be effectively suppressed. There have been relatively few studies on the manner in which laser peening acts on BMGs; the effects of duration on SB formation and growth remain unclear.

This paper reports an extra-deep shock-affected zone (about 3500 μm) formed via nanosecond laser peening, that is much larger than the one in conventional crystal metal (less than 2000 μm). Numerous arc SBs dozens of micrometers in length are observed in this region, the characteristics and corresponding energy dissipation mechanism of which are discussed in detail below.

## Results

**Figure 1:**
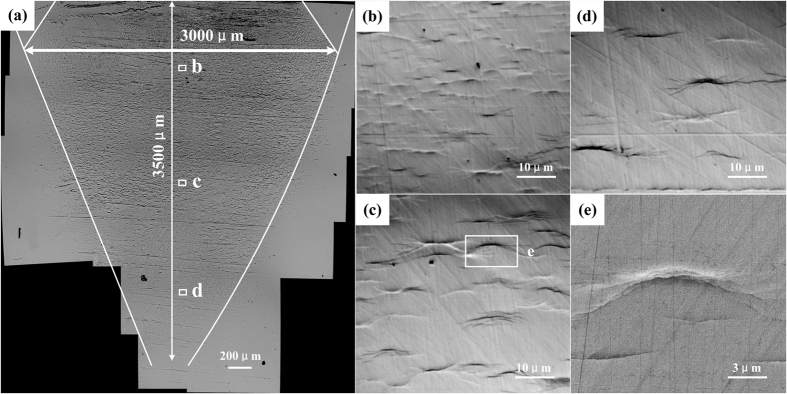
(**a**) Shock-affected zone in Vit 1 BMGs; (**b**) area near the surface; (**c**) central affected area away from the surface; (**d**) area at the end of the shock-affected zone; (**e**) enlarged arc SB in (**c**).

Irrecoverable plastic deformation occurs if the laser-induced shock pressure is above the dynamic yield strength of the target material. The plastic-affected zone of Vit1 BMGs along the direction of the laser beam is shown in Fig. 1a. A tapered affected region 3500 μm in depth and 3000 μm in width was clearly observed in the central portion of the focused laser spot. The width of the affected zone was smaller than the initial laser spot because the shock pressure at the edge of the spot was too low to cause BMGs to yield. Likewise, the deepest observable deformation zone was located at the very center of the shock wave where pressure was at its maximum.

The changes in BMG microstructure at different peened layer depths are also described in Fig. 1. Numerous arc SBs 5–30 μm in size appeared and aggregated in the vertical direction of the laser beam, which is different from the conventional full-length SBs^12^,^13^,^14^,^15^,^16^,^17^,^18^,^19^,^20^,^21^,^22^. As shown in Fig. 1b, the arc SB exhibited short and discrete features near the surface of the material. It then grew longer and fewer at deeper peened layer depths (Fig. 1c,d). SB development is an important mode of localized inhomogeneous deformation that occurs in BMGs, however, the onset and evolution of arc SB upon laser peening had not been explored prior to this study.

**Figure 2:**
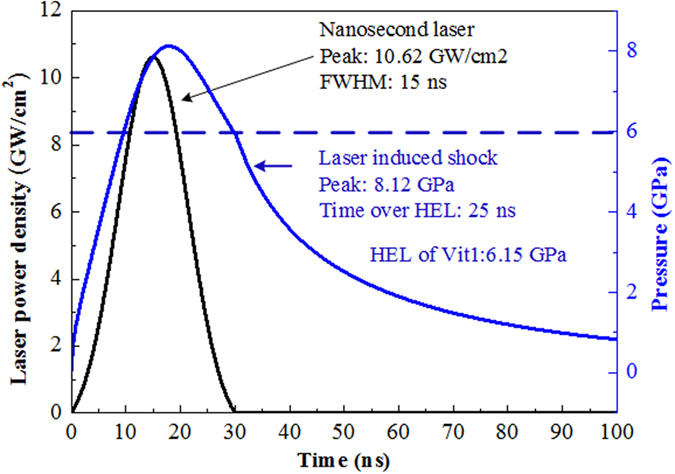
Relationship between power density and shock pressure during laser shock peening.

Laser power density and calculated shock pressure profiles were obtained in order to reveal the formation and evolution of the arc SB more clearly. The SB can be activated if the shock pressure exceeds the Hugoniot elastic limit (HEL). For materials with low toughness, plastic deformation will occur if the pressure it suffered is higher than HEL. The experimental HEL value of Vit1 BMG is about 6.15 GPa^32^. As shown in Fig. 2, the laser-induced shock pressure is characterized at first by large shock peening and short action time near the surface. This shock pressure may last dozens of nanoseconds (80–90 *ns*). The duration above HEL is only about 25 *ns*, however, which does not leave sufficient time for SB full propagation. If we assume that the SB front propagates in the form of a shear wave, the propagation of SBs has been suspended instantaneously upon onset, relating only to the very early stages of shear-band initiation. Actually, the simultaneous and progressive shear models^33^ depend on the action time of SB, if the action time is very small in ~ ns, then it is simultaneous compared with action time of ms scale. Therefore, immature SBs with dozens of micrometer in length are obtained before the band can collectively slip as a whole. In addition, the shock energy attenuates in BMGs owing to the formation of SBs. The shock pressure profile weakens in amplitude and stretches across time corresponding to an increased length and decreased number of SBs at deeper peened layer depths.

**Figure 3:**
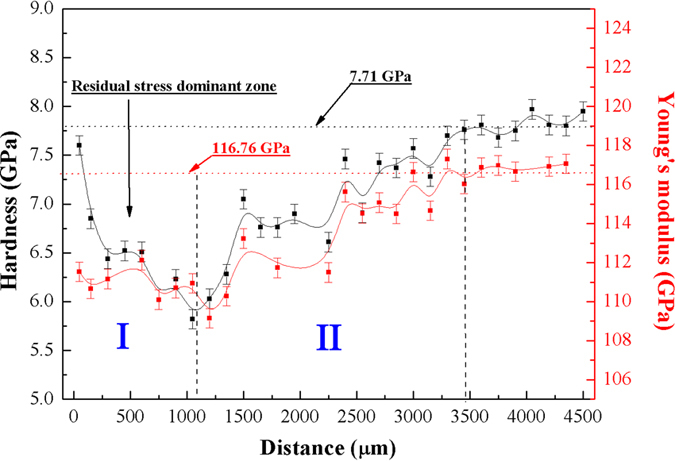
Distributions of hardness (Black) and Young’s modulus (Red) along the laser shock wave direction.

The mechanical behavior of Vit 1 BMGs is closely related to arc SB evolution. As shown in Fig. 3, the Young’s modulus *E* is lower than the matrix near the surface due to arc SB aggregation, exhibiting a work-soften phenomenon. In the end of the shock-affected zone, conversely, the Young’s modulus *E* increases likely corresponding to the disappearance of arc SBs^34^. Interestingly, the changes in hardness are more complex owing to the combine effect of both the induced residual stress and the distribution of arc SBs. Along the depth direction, the compressive residual stress gradually reduced to zero, then change to the tensile residual stress. The tensile stress initially increases, and then rapidly decreases to zero^35^. The presence of the compressive residual stress near the surface can boost up the apparent hardness. For force balance, the tensile residual stress in the deeper impacted region can decrease the hardness. Nieh *et al*. proved that hardness gradually decreases to its minimum value as the depth increases^36^, corresponding to the residual stress dominant zone. As show in Fig. 3, the minimum value in the hardness curve appears at the depth between 1100 μm and 1200 μm, suggesting that the depth of the residual stress dominant zone is likely between 1100 μm and 1200 μm.

In addition, the apparent hardness can be efficiently modified associated with the formation of abundant arc SBs, which typically leads to BMG softening phenomena^29^,^37^. In the second half of the hardness curve (Region II), the SBs play a dominant role in the softening of BMGs compared with the residual stress. The number of SBs becomes less, which corresponds to the rejuvenation of hardness. The hardness in the shock affected region is smaller than that in the matrix region, and many dips or local extremes appear in the profile owing to the combined effect of both the induced residual stress and SBs.

## Discussions

The energy dissipation density is an apparent parameter which reflects the ability of materials for accommodating the applied shear strain and plastic deformation during the laser shock peening. In crystal metal, the fundamental structural unit for accommodating plastic deformation is viewed as the pre-existed defects, which are widely distributed and easily activated by the high speed shock. However, owing to the lack of defects in BMGs, SBs may serve as the basic unit to accommodate plastic deformation, analogous to dislocations in crystalline materials^12^,^13^,^14^,^15^,^16^,^17^,^18^,^19^,^20^. This is to say, SBs may also play a dominant quantitative role in energy dissipation. If energy input that induces the plastic deformation is constant, this larger affected volume usually leads to the smaller energy dissipation density for BMG compared to crystalline metal. How does the discrete arc SB accommodate the plastic deformation upon laser peening? The answer to this question may lie in an energy dissipation model such as the one discussed below, which was established based on the HEL as well as SB characteristics.

**Figure 4:**
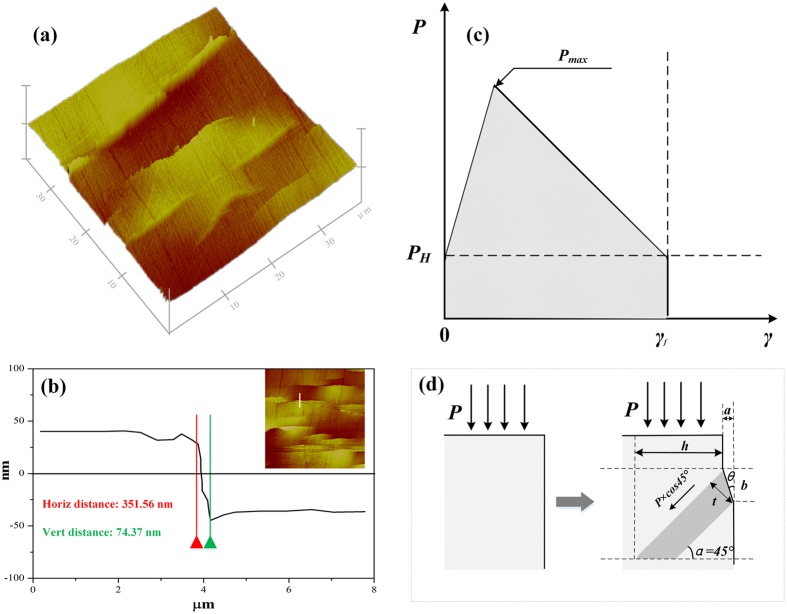
AFM micrographs of Vit1 BMG surfaces: (**a**) surface morphology; (**b**) cross-sectional profiles; (**c**) and (**d**) schematic diagrams of energy dissipation and arc SB formation.

As shown in Fig. 4, a 45° shear direction and triangular shock pressure were selected to simplify the calculations. The average energy dissipation density of BMGs can be expressed as follows:


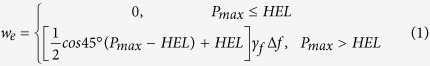


where *P*_*max*_ is the peak pressure at certain location along the central line; *γ*_*f*_ = *a/b* is the average ultimate shear strain, and *a* and *b* can be obtained from the AFM shown in Fig. 4b; ∆f = V^*^/*V* is the volume percentage that participates in shearing^38^; 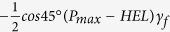 represents the energy dissipation in a local arc SB. In order to obtain *∆f*, 13 typical regions with *A* = *75* × *75* *μm*^*2*^ were chosen in the central portion of the peened layer to determine the number *n* and an average arc length *l*. The thickness of SB *t* can be calculated as follows:


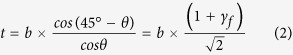


So ∆*f* is:


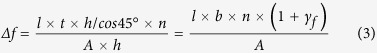


**Figure 5:**
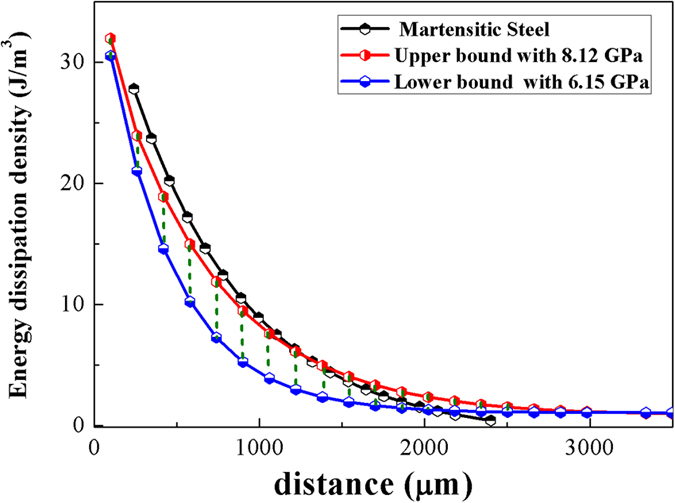
Energy dissipation density curves in Vit1 BMGs (green region) and martensitic steel (black line).

As mentioned above, the pressure produced by nanosecond peening is not constant. It decreases from *P*_*max*_ (8.12 GPa) to *P*_*min*_ (6.15 GPa) as the shock wave attenuates. The attenuation curve cannot be predicted because the interaction between the BMGs and laser peening is unclear. The interzone energy dissipation density between *P*_*max*_ and *P*_*min*_ can be obtained, however, using Eq. (1). As shown in Fig. 5, the interzone energy dissipation density of BMGs was lower than that of martensitic steel in most regions, suggesting that there was slower energy dissipation in the BMGs. The energy dissipation density essentially depends on pressure-induced strain in the martensitic steel, in which the dislocation or twin crystal can be viewed as the basic unit to accommodate the slide and energy dissipation^38^,^39^. Owing to the lack of defects, only the arc SBs contribute to the plastic deformation in BMGs, which is main cause of the ultra-deep affected zone that formed in Vit 1 BMG samples.

In conclusion, a 3500 μm-deep affected zone was observed in Vit1 BMGs upon laser peening. As opposed to the conventional SBs, numerous arc SBs appeared and aggregated in the vertical direction of the laser beam; the bands acted as basic units for accommodating plastic deformation. The arc SBs exhibit short and discrete features near the surface of the material, then grow longer and fewer at deeper peened layer depths, in accordance with the attenuation of laser shock wave. BMGs are found to possess a lower energy dissipation density than crystal metal, which have a close relationship with the formation of an extra-deep shock-affected zone. The results reveal that nanosecond laser peening is promising as an effective approach to tuning SBs for improved MGs ductility.

## Methods

**Figure 6:**
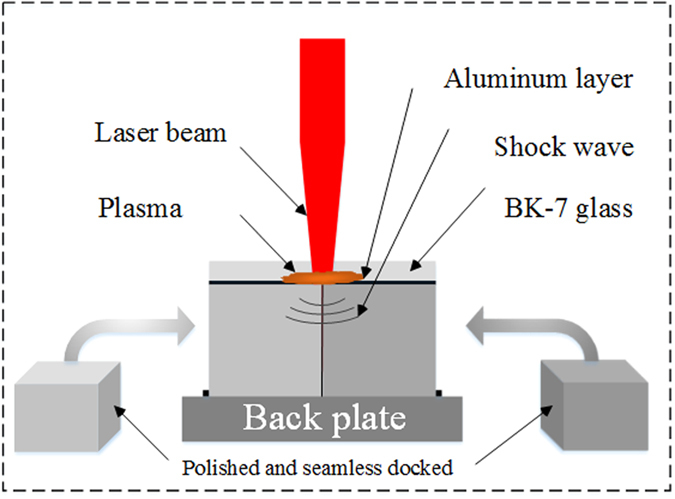
Schematics of the laser induced shock experiments for BMG.

Zr_41_Ti_14_Cu_12.5_Ni_10_Be_22.5_ BMG was chosen as the target material. Two target specimens (5 × 5 × 5 mm^3^) were cut by wire electrical discharge machining of the as-cast material and two adjacent surfaces were polished to remove oxides. The polished surfaces were seamlessly docked together as shown in Fig. 6. A 50 μm-thick self-adhesive aluminum foil was used as an overlay to eliminate ablation on the top surface and a 4mm thick BK-7 glass was placed on top of the aluminum foil as a confined layer to constrain the plasma generated by the laser irradiation. Another Vit1 plate was placed at the bottom to ensure that waves were not reflected back into the specimens. A 12 mm-diameter laser pulse was generated by a Q-switched Nd:YAG laser (wavelength 1064 nm, pulse width 15 ns, and pulse energy 15 J) and focused to 4.5 mm once arriving on the target surface. The profiles of laser power density and calculated shock pressure^30^ were as shown in Fig. 2. Before and after shock, X-ray diffraction (XRD) analysis was carried out on a Philips PW 1050 diffractometer using CuKα radiation. The morphology was observed on a scanning electron microscope (SEM, JEM-2100F) and atomic force microscope (AFM, Hysitron Tribo Scope).

## Additional Information

**How to cite this article:** Wei, Y. *et al*. Anomalous shear band characteristics and extra-deep shock-affected zone in Zr-based bulk metallic glass treated with nanosecond laser peening. *Sci. Rep.* **7**, 43948; doi: 10.1038/srep43948 (2017).

**Publisher's note:** Springer Nature remains neutral with regard to jurisdictional claims in published maps and institutional affiliations.
